# Medicinal Honeys from Oceania: An Updated Review on Their Bioactive Constituents and Health Applications

**DOI:** 10.3390/biotech15010005

**Published:** 2026-01-12

**Authors:** Maryna Lutsenko, Michela Ravelli, Gregorio Peron

**Affiliations:** 1 Department of Molecular and Translational Medicine, University of Brescia, 25123 Brescia, Italy; maryna.lutsenko@unibs.it (M.L.); ravellimichela5@gmail.com (M.R.); 2 Department of Professional Education, Restaurant and Tourism Business, Taras Shevchenko Luhansk National University, 36000 Poltava, Ukraine

**Keywords:** honey, antimicrobial, antioxidant, Manuka, Jarrah, Agastache

## Abstract

Medicinal honeys from Oceania have gained considerable attention due to their peculiar bioactive constituents and potential health applications. Apart from small molecules such as methylglyoxal and hydrogen peroxide, these honeys are rich in phenolic compounds, volatile terpenes, and other bioactive molecules, which collectively contribute to their antioxidant, antimicrobial, anti-inflammatory, and wound-healing properties. Recent studies have highlighted the distinctive composition of Oceania honeys such as Manuka (*Leptospermum scoparium*), Jarrah (*Eucalyptus marginata*), and Agastache (*Agastache rugosa*) from New Zealand and Australia, demonstrating variability in bioactivity depending on floral source, geographical origin, and processing methods. This review synthesizes the current knowledge on the chemical profiles of these honeys with a particular focus on bioactive compounds and distinctive markers, and evaluates their therapeutic potential. Emphasis is placed on the mechanisms underlying their bioactivities, as well as emerging clinical and preclinical evidence supporting their medicinal use. By consolidating recent findings, this work provides an updated perspective on the functional properties of Oceania honeys, underscoring their relevance as natural products with significant health-promoting potential.

## 1. Introduction

The term “honey” refers to the natural, sweet substance produced by *Apis mellifera* and other bee species from the nectar of flowers or the secretions of living plant parts, which they collect, transform, enrich with their own specific substances, and subsequently store and ripen in the comb [1,2]. As a globally traded commodity, its definition and quality standards are legally defined by regulatory bodies such as the Codex Alimentarius [3] and the EU Council Directive on honey [4]. Honey is broadly categorized by origin into floral (nectar-based) and honeydew (produced from insect secretions on plants) [5]. While historical accounts dating back over 8000 years highlight its traditional role as a food, ritual offering, and medicinal agent due to its antimicrobial and wound-healing properties [6,7], its modern value lies in its complex chemical composition and associated bioactive potential.

The botanical and geographical origin is the single most critical factor determining honey’s composition, quality, and market value [8]. Verifying this origin is essential for controlling food fraud and guaranteeing product efficacy, driving the adoption of diverse methodological approaches, including the determination of sugar profiles, amino acid profiles, protein content, and phenolic compound profiles [9]. Traditionally, melissopalynological analysis (the study of pollen content) has been the primary method for origin determination, but it suffers from significant analytical limitations [10]. The pollen count is not exclusively related to the source nectar (primary enrichment) but is confounded by secondary enrichment (bee processing), tertiary enrichment (handling and extraction), and quaternary enrichment (airborne dispersion) [11]. Furthermore, factors such as hive migration and the need for highly specialized personnel and stringent method standardization render pollen analysis often inconclusive for definitive origin verification. This analytical ambiguity creates a critical gap in authentication practices.

To overcome these analytical limitations and provide a more robust basis for both authentication and therapeutic assessment, attention has shifted towards the non-volatile chemical fraction, specifically the polyphenols. Polyphenols are considered promising markers of honey authenticity because their content is significantly influenced by the botanical source, acting as a unique chemical fingerprint [12,13]. The application of High-Performance Liquid Chromatography combined with Mass Spectrometry (LC-MS) is now fundamental, allowing for the comprehensive separation and identification of complex phenolic profiles based on structure and mass [14].

In recent years, research has increasingly focused on honeys produced from distinctive botanical and geographical contexts, where unique floral sources give rise to specific chemical profiles and bioactive properties. Among these, honeys from Oceania including monofloral varieties from New Zealand and Australia have attracted considerable attention due to their particular composition in bioactive compounds and demonstrated antimicrobial, antioxidant, anti-inflammatory, and wound-healing activities. These findings highlight the potential of Oceania honeys as medicinal natural products and provide a strong rationale for their systematic evaluation.

The aim of this review is to provide a critical and structured overview of medicinal honeys from Oceania by integrating information on their chemical composition, bioactive constituents, and region-specific pharmacological evidence. Following a general examination of honey’s chemical and nutritional properties, the review focuses on selected monofloral honeys from New Zealand and Australia, highlighting how botanical origin influences the presence of distinctive chemical markers and bioactive compounds. Particular attention is given to mechanistic insights and preclinical and clinical evidence supporting their therapeutic potential.

## 2. Literature Search and Selection Methodology

The literature included in this review was identified through a structured search strategy following general PRISMA guidelines for narrative reviews. Electronic searches were conducted in major scientific databases, including Scopus and PubMed, covering publications up to 2025. Additional relevant articles were identified by screening the reference lists of key reviews and original research papers. The search strategy combined keywords related to honey composition and bioactivity with terms specific to Oceania honeys. Search terms included combinations of “honey”, “medicinal honey”, “Manuka”, “Jarrah”, “Leptospermum”, “Eucalyptus”, “Agastache”, “Oceania”, “Australia”, “New Zealand”, “polyphenols”, “chemical composition”, “authentication”, “antimicrobial”, “antioxidant”, “anti-inflammatory”, and “wound healing”. Boolean operators (“AND”, “OR”) were used to refine searches. Studies were included if they (1) reported original experimental, preclinical, or clinical data, or constituted relevant review articles; (2) focused on honeys specifically originating from Oceania; and (3) provided information on chemical composition, bioactive constituents, authentication markers, or biological activity. Exclusion criteria comprised studies not specifying botanical or geographical origin, articles lacking primary analytical or biological data, and non-peer-reviewed sources.

The review primarily considers studies published between 2019 and 2025. This period was selected because foundational studies characterizing honeys from botanical sources such as *Agastache* spp. and comprehensive comparative analyses including Jarrah honey were first published around 2019. Subsequent research has meaningfully expanded the field by providing new analytical insights, emerging composition-function relationships, and additional pharmacological evidence. Focusing on this recent period differentiates the present review from earlier general honey reviews and emphasizes recent progress, particularly for less-studied honeys such as Agastache and Jarrah.

## 3. Chemical and Nutritional Composition of Honey

Honey is a complex matrix composed of over 180 identified compounds, the relative concentrations of which are intrinsically determined by botanical and geographical origin, environmental conditions, bee species, and post-harvest processing methods [15,16]. This compositional variability dictates differences in its physical attributes, including color, viscosity, flavor, and bioactivity [15]. Honey typically consists primarily of carbohydrates (approximately 75–80%) and water (17–20%), with a minor, yet functionally essential, fraction (<5%) comprising non-sugar components such as proteins, enzymes, amino acids, polyphenols, and minerals (Table 1) [17,18].

### 3.1. Carbohydrates

The predominant constituents of honey are monosaccharides, specifically fructose (typically 38–41%) and glucose (31–35%), which collectively account for over 85% of the total sugar content [19]. These simple sugars are formed through the enzymatic hydrolysis of sucrose catalyzed by invertase (or sucrase) secreted by the bees during the trophallaxis process [20,21]. The ratio and concentration of these sugars significantly influence honey’s primary physicochemical characteristics, including its water activity, crystallization rate, viscosity, and rheological behavior [22,23], but also its nutritional properties.

Minor carbohydrate fractions include disaccharides and oligosaccharides (typically <10%). While maltose and sucrose are the most common disaccharides, over a dozen others (e.g., turanose, kojibiose, melibiose) have been identified. A significant proportion of oligosaccharides (trisaccharides, tetrasaccharides, and pentasaccharides) are neo-formed and are not directly derived from nectar. Their origin is attributed to trans-D-glycosylation reactions and non-enzymatic acid reversion (condensation of monosaccharides), both favored by the high sugar concentration and acidic pH of the honey matrix [24,25,26,27,28,29].

Given that carbohydrate profiles can vary significantly with botanical origin, their determination (commonly performed by HPLC, gas chromatography (GC), and MS [30]) is a robust tool. This analytical approach is critical for detecting adulteration, specifically the addition of exogenous syrups (e.g., corn, rice) which exhibit distinct sugar profiles compared to natural honey.

### 3.2. Phenolic Compounds

The non-sugar fraction of honey is fundamental to its bioactivity. Polyphenols, which are secondary plant metabolites, are primarily transferred to honey through flower nectar, pollen, and propolis [31]. These compounds significantly contribute to the honey’s color, flavor, and, most importantly, its antioxidant capacity [13]. The two primary classes of polyphenols in honey are flavonoids and phenolic acids [31].

#### 3.2.1. Flavonoids

Flavonoids represent the largest and most structurally diverse class of phenolic compounds, characterized by a C_6_-C_3_-C_6_ flavane nucleus. Their inherent diversity arises from structural modifications such as hydroxylation, *O*-methylation, dimerization, and glycosylation reactions [32,33], which classify them into subclasses including flavonols, flavones, flavanones, and anthocyanidins [33,34]. In honey, the total flavonoid content can reach up to 6 mg/kg [35], with common examples including pinocembrin, apigenin, quercetin, galangin, and pinobanksin [13,36]. The specific qualitative and quantitative profile of flavonoids serves as a highly reliable chemical biomarker for determining the botanical origin of honey.

Therapeutic relevance of flavonoids stems from their well-documented anti-inflammatory, antimicrobial, antiallergic, antiviral, antioxidant, and cytotoxic activities. For instance, the 5,7-dihydroxyflavanone pinocembrin exerts anti-inflammatory, antifibrotic, and antioxidant activities [37,38,39,40,41], while quercetin and galangin exert a wide antimicrobial effect against bacteria, viruses and fungi of clinical interest, as demonstrated by several studies [42].

#### 3.2.2. Phenolic Acids

These compounds, derived from either benzoic acid or cinnamic acid, are also crucial components originating from plant nectar [43,44,45,46,47,48,49]. They exist predominantly in ester, amide, or glycosidic forms [50] Common derivatives of benzoic acid include *p*-hydroxybenzoic, protocatechuic, and vanillic acids, while cinnamic acid derivatives include caffeic, *p*-coumaric, ferulic, and sinapic acids [51] The phenolic profile of honey depends on the botanical and geographical origin and contributes to its antioxidant capacity, together with flavonoids and enzymatic antioxidants. Common antioxidants in honey include gallic acid, caffeic acid, coumaric acid, and chlorogenic acid [13,48].

The antioxidant capacity of honey is significantly dependent on the presence of phenolic compounds. A 2020 study found a significant correlation between phenolic content, antioxidant capacity, and color. The total phenolic acid content of the honeys analyzed ranged from 3.57 to 18.83 mg CAE/100 g (concentration of phenolic acids in 100 g of honey), with the highest content observed in buckwheat honey and the lowest in clear honey [52].

### 3.3. Minor Components: Organic Acids, Proteins, and Minerals

Organic acids (OAs) represent a small but critical fraction that profoundly influences honey’s flavor and long-term stability, due to its resulting low pH (typically 3.2 to 4.5) [53]. Gluconic acid is the most predominant organic acid, formed via the oxidation of glucose by the enzyme glucose oxidase, a process integral to honey maturation. Other OAs, such as lactic, acetic, and citric acids, may originate from enzymatic synthesis by bees, plant secretions, or microbial fermentation [54,55,56].

Honey contains trace amounts of proteins and amino acids (e.g., proline, arginine), primarily derived from glandular secretions of the bees [19]. Key protein components are the enzymes, including: invertase (sucrase), which hydrolyzes sucrose into glucose and fructose; diastase (amylase), which breaks down starch; catalase and phosphatase. Enzyme activity, particularly diastase activity, is a globally recognized quality parameter used to verify that honey has not been subjected to excessive heat treatment (which denatures enzymes) [31].

Minerals, though typically less than 1% of the composition, are highly variable; darker honeys usually contain higher concentrations [16]. Potassium is the most abundant element (45–85% of total minerals), followed by calcium, sodium, magnesium, and trace elements like zinc, iron, and copper [16,57]. The mineral content not only impacts nutritional value but also serves as a partial geo-marker for origin determination.

Other minor compounds can be determined in honey as contaminants. The presence of heavy metals (e.g., cadmium, lead, arsenic) or pesticide residues acts as a direct bioindicator of environmental pollution in the forage area where the bees collected nectar [58]. Other compounds can be derived from processing. The most critical marker of poor handling or excessive heating is 5-hydroxymethylfurfural (HMF). HMF is a six-carbon heterocyclic compound that forms via the Maillard reaction from reducing sugars in acidic environments or through sugar self-degradation [57]. High HMF levels are universally regulated by quality standards (e.g., Codex Alimentarius, EU Directive) as an indicator of freshness and quality maintenance during processing and storage [59].

## 4. Therapeutic and Pharmacological Properties of Honey

The application of beehive products for therapeutic purposes, known as apitherapy, is experiencing a resurgence in evidence-based medicine [60]. Honey, in particular, exhibits a multifaceted spectrum of biological activities driven by its complex chemical matrix. The most compelling activities, explored below, include its potent antioxidant, antimicrobial, anti-inflammatory, and emerging antitumor potential.

### 4.1. Antioxidant Activity

Several components of honey such as ascorbic acid, carotenoids, and enzymes (e.g., glucose oxidase and catalase) have antioxidant properties and can act synergically. However, phenolic compounds have been reported as the main responsible of the antioxidant property [34]. The botanical and geographical origin is the paramount factor determining antioxidant capacity, with a strong correlation observed between the total content of phenolic acids and overall antioxidant activity [7]. Darker honeys are generally believed to contain higher concentrations of these phenolics, linking color to antioxidant potential [7]. A key advantage of honey is its lack of a pro-oxidant effect when administered therapeutically, a phenomenon sometimes observed with high doses of isolated classic antioxidants like vitamins C and E [61,62]. This protective effect is attributed to the complex interplay of multiple compounds present in the honey matrix. The systemic benefits of honey’s antioxidant action are supported by preclinical models: in rats, pretreatment with wild honey protected against epinephrine-induced cardiac disorders and vasomotor dysfunction, highlighting its cardioprotective effects [63]. Tualang honey demonstrated a hypoglycemic effect in diabetic rats while improving renal oxidative stress [61]. The ester derivatives of caffeic acid (present at 20–25% in honey) are thought to contribute to its cytotoxic activity, with in vitro studies confirming significant inhibition of human hepatocellular carcinoma (HepG2) cell line proliferation [64].

### 4.2. Antimicrobial Activity

Honey’s capacity to counteract various pathogenic organisms is attributed to several mechanisms, including its high sugar content and low pH (creating an inhospitable environment), the controlled enzymatic production of hydrogen peroxide, and the presence of non-peroxide components such as polyphenolic compounds and antimicrobial peptides [65,66].

The therapeutic use of honey is gaining traction, particularly for infections caused by antibiotic-resistant pathogens like Methicillin-Resistant *Staphylococcus aureus* (MRSA) [67,68]. Its broad-spectrum efficacy, demonstrated in vitro, positions it as a potential adjunct treatment to combat the rising threat of bacterial resistance [68].

Regarding honey’s activity against fungi, in vitro efficacy has been reported against fluconazole-resistant fungal infections, such as those caused by *Candida albicans* in immunocompromised patients, suggesting a future role against drug-resistant fungal strains [31,69].

Finally, regarding antiviral activity, honey has demonstrated the ability to accelerate the healing time of skin lesions caused by HSV compared to standard antiviral agents like acyclovir [70].

### 4.3. Anti-Inflammatory and Wound Healing Activity

Honey’s anti-inflammatory properties are linked to its bioactive constituents, notably flavonoids, which modulate the expression of pro- and anti-inflammatory cytokines (e.g., TNF-alpha, IL-2, IL-10, IL-12p70, and Interferon-gamma) [71].

Flavonoid extracts from honey have been shown to reduce the production of pro-inflammatory mediators in activated microglia, suggesting a potential role in attenuating the neuroinflammation associated with neurodegenerative diseases like Alzheimer’s and Parkinson’s [72]. Honey inhibits the generation of ROS and can modulate cyclooxygenase-2 activity [73]. Clinical administration of honey in healthy adults has been shown to reduce plasma concentrations of key inflammatory markers, including thromboxane B2, PGE2, and PGF2-alpha, supporting its use as an adjunct anti-inflammatory agent [73].

Honey is widely recognized in wound care. A seminal clinical study comparing honey to silver sulfadiazine for burn wounds found that honey treatment resulted in faster epithelialization, earlier resolution of acute inflammation, and improved infection control [74]. This efficacy is due to its physical barrier function combined with its antimicrobial and anti-inflammatory effects [75]. Evidence suggests also that honey may stimulate the proliferation and differentiation of stem cells, indicating a potential role in regenerative medicine [76,77].

### 4.4. Antitumor Activity

Honey exhibits promising antitumor properties by modulating critical cellular mechanisms, primarily apoptosis and cell proliferation [21,31]. In vitro studies show that honey depolarizes the mitochondrial membrane and activates the intrinsic apoptotic pathway by stimulating the expression of pro-apoptotic factors (e.g., p53 and caspases 3 and 9) while reducing anti-apoptotic factors (e.g., Bcl-2) [31]. Oral co-administration of honey and *Aloe vera* has been shown to decrease the nuclear protein Ki-67, a marker present during cell proliferation phases. This reduction slows the division rate of cancer cells, thereby inhibiting tumor growth [31].

Honey can activate the immune system to counteract tumor growth, and specifically proteins such as apalbumin-1 and apalbumin-2 can induce the release of TNF-alpha from macrophages [78,79,80,81]. The molecular mechanisms underlying these effects include the modulation of oxidative stress, reduction in inflammation, and inhibition of angiogenesis in tumor cells [21]. While preclinical evidence is compelling, scientific data on honey’s efficacy as a primary antitumor agent is currently limited to cellular studies, necessitating further investigation in more complex in vivo and clinical models to fully confirm its therapeutic potential [31]. The path forward requires targeted research to link the chemical composition of specific therapeutic honeys to their defined pharmacological outcomes.

## 5. Chemical and Pharmacological Properties of Oceanian Medicinal Honeys

This section shifts the focus to an in-depth analysis of high-value medicinal honeys from the Oceania region, specifically Manuka, Agastache, and Jarrah honeys. The distinct therapeutic profiles of these honeys are fundamentally driven by unique, non-peroxide chemical components.

### 5.1. Manuka Honey

Manuka honey, derived from the nectar of *Leptospermum scoparium* (Myrtaceae), is a dark, monofloral honey primarily produced in New Zealand and Australia [82,83,84,85,86,87]. It is globally recognized for its unique characteristics, including a distinctive earthy flavor, variable color (deep cream to amber), and a robust, non-peroxide-based antibacterial activity [88,89,90].

The phytotherapeutic use of Manuka honey is largely attributed to methylglyoxal (MGO), a reactive alpha-oxoaldehyde [91]. MGO is spontaneously formed during honey maturation from its precursor, dihydroxyacetone (DHA), which accumulates in high concentrations specifically in the nectar of *L. scoparium* [92]. The formation of MGO is catalyzed by the unique physiological environment of the honey: DHA, present in the nectar, is converted to MGO during the maturation period, driven by the actions of the bees and time [93,94,95,96]. The conversion rate is influenced by factors such as storage conditions and moderate heat, which can be intentionally utilized post-harvest to increase MGO concentration, thereby enhancing the antibacterial efficacy [97]. The concentration of MGO in Manuka honey typically ranges significantly, from 30 to over 800 mg/kg, and is linearly correlated with its unique and potent antibacterial activity [91]. While MGO is not exclusive to Manuka honey (being detected at substantially lower concentrations in other varieties, such as 0.4–17 mg/kg in some Italian honeys [98,99]), its high concentration remains the definitive chemical marker.

**Figure 1:**
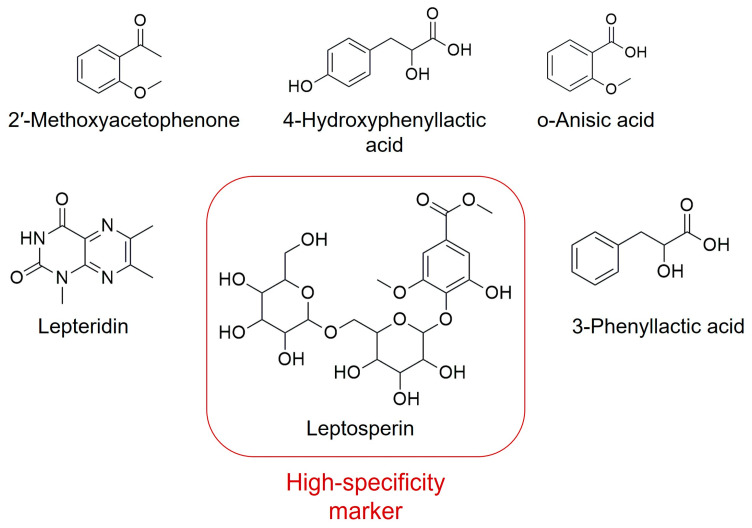
Chemical markers of Manuka honey.

The quality and efficacy of Manuka honey are standardized using the Unique Manuka Factor (UMF), a standardized scoring system developed in New Zealand [91]. The UMF is a non-physical numerical index that primarily reflects the concentration of MGO (and its precursor DHA), in addition to other compounds like leptosperin [91]. Higher UMF ratings indicate a stronger overall antibacterial potency, effective against both Gram-positive and Gram-negative pathogens [91]. To ensure product authenticity and distinguish Manuka from botanically similar honeys (e.g., Kanuka, Kunzea ericoides), specific chemical markers are utilized alongside MGO, including leptosperin, lepteridine, 3-phenyllactic acid, 4-hydroxyphenyllactic acid, 2-methoxybenzoic acid (*o*-anisic acid), and 2′-methoxyacetophenone (Figure 1). Some of these compounds have been detected also in other medicinal honeys, although in lower amounts and different ratios. Leptosperin has been specifically highlighted as a unique chemical marker identified in Manuka honey. These compounds have also been associated with the antimicrobial properties of Manuka honey [90]. Furthermore, advanced palynological techniques allow for the differentiation of *L. scoparium* pollen from *K. ericoides* based on morphological distinctions like equatorial diameter and surface texture [100].

In addition to these constituents, phenolic acids such as protocatechuic acid, syringic acid, and gallic acid are present, as well as flavonoids such as quercetin, luteolin, apigenin, kaempferol, galangin, pinobanksin, and pinocembrin. The total phenolic content (TPC) of manuka honey samples ranges from 250 to 985 µg/g, depending on the geographic origin and storage and processing conditions. Among the identified phenolic acids, gallic acid represents approximately 49% of the total phenolic acids, followed by syringic acid which represents 44%. Among the flavonoids, quercetin represents 46% of the total content, luteolin 33% and kaempferol 15%.

Among the vitamins, the most represented are thiamine (B1), riboflavin (B2), ascorbic acid (vitamin C), pantothenic acid and pyridoxine. Vitamin C in this type of honey has a concentration equal to 1067.4 mg/kg. All these phytocompounds are responsible for the antimicrobial, antioxidant, anti-inflammatory, anti-tumor, immunomodulatory and wound healing properties of manuka honey [89]. Based on this distinctive chemical composition, Table 2 summarizes representative in vitro data on the antibacterial activity of Manuka honey against clinically relevant microorganisms.

Consistent with this distinctive chemical profile, Manuka honey exhibits potent antimicrobial activity, which has elevated its status from a “superfood” [101] to an approved Medical-Grade Honey (MGH) used clinically, particularly in advanced wound and burn care. MGH is subject to stringent quality and safety standards for medical use [91]. In compliance with international regulations (e.g., European Directive 2001, updated 2019, and Regulation (EU) 2017/745), MGH must be sterile, organic, and free of contaminants [101,102,103]. Sterility is typically achieved through gamma irradiation [102]. While this process may cause a minor reduction in antimicrobial potency, it is essential for ensuring microbiological safety in the clinical setting, allowing MGH to be governed under medical device regulations due to its proven wound management applications.

The antimicrobial effect is quantitatively assessed using the Minimum Inhibitory Concentration (MIC), defined as the lowest concentration of the honey or its pure compound (e.g., MGO) required to inhibit visible microbial growth [91]. For pure MGO, MIC values of 0.15–0.31 mg/mL have been reported against common pathogens such as *S. aureus* and *P. aeruginosa*.

#### 5.1.1. Optimizing Bioactive Content: Correlation Between Flower Development and Metabolite Production

Optimizing manuka honey production requires maximizing nectar sugar levels while preserving high concentrations of DHA. This can be achieved through increased floral abundance or enhanced nectar volume, both of which are influenced by environmental factors such as leaf area and temperature, which govern sugar biosynthesis. Smallfield (2018) [90] classified eight distinct floral developmental stages (FDS), ranging from immature buds to post-anthesis flowers with wrinkled stamens and nectar-rich hypanthia. Total sugars and DHA progressively increased across development, peaking immediately before floral senescence. Authenticity markers of manuka honey, including leptosperin, lepteridine, and 3-phenyllactic acid, exhibited similar accumulation patterns, indicating biosynthetic linkage with nectar production [90].

Metabolite-specific trends were also observed: 2′-methoxyacetophenone reached maximal levels at stage 5, whereas 2-methoxybenzoic acid increased continuously throughout anthesis. In contrast, leptosperin and lepteridine peaked earlier than methylsyringate and 2-methoxybenzoic acid [90]. These findings demonstrate that the floral development stage is a critical determinant of nectar composition and, consequently, honey quality.

#### 5.1.2. Antimicrobial and Wound Healing Properties of Manuka Honey

Manuka honey has significant antibacterial activity [103]. The efficacy against *S. aureus* has been evaluated in comparison with sidr honey (SH) and tualang honey (TH) using agar diffusion, MIC, minimum bactericidal concentration (MBC), time-kill curve, microtiter plate, and RT-qPCR assays. All honeys demonstrated inhibitory activity, with Manuka exhibiting the largest inhibition zones [104]. MIC and MBC values confirmed greater potency for Manuka (12.5% and 25% *v*/*v*, respectively) compared to SH and TH (MIC: 25%, MBC: 50%). Biofilm formation was significantly reduced at 60% honey concentration, with Manuka achieving the highest inhibition (52%), followed by SH (46%) and TH (42%). Time-kill curves revealed reduced colony-forming units after honey exposure, while RT-qPCR demonstrated downregulation of multiple *S. aureus* virulence genes, with Manuka showing the strongest effects [104].

In the clinical context of wound management, the topical application of honey is beneficial for maintaining a moist healing environment, providing a protective barrier against exogenous and endogenous microbial contamination, and accelerating tissue repair [104]. This therapeutic action is enhanced by the relatively low pH (3.5–4.5) of Manuka honey, which contributes to antibacterial activity, promotes fibroblast activation and tissue oxygenation, and aids in mitigating tissue damage associated with ROS by limiting inflammation [103]. Antimicrobial efficacy can also be leveraged synergistically, as studies have reported positive Fractional Inhibitory Concentration Index (FICI) values when Manuka honey is combined with conventional antibiotics like tetracycline and oxacillin [105,106].

Despite its compelling clinical efficacy, the inherent viscosity and limited mechanical stability of raw honey present challenges for consistent topical dosing and application [107]. To address this, biomedical engineering approaches have focused on incorporating Manuka honey into advanced scaffold and hydrogel systems. For instance, researchers have successfully developed three-dimensionally printed honey-gelatin hydrogel patches that not only retained antibacterial properties but also actively stimulated fibroblast and keratinocyte proliferation and promoted angiogenesis [107]. Similarly, composite systems, such as 2-hydroxyethyl methacrylate/gelatin (HG) hydrogel scaffolds, have improved structural integrity, swelling behavior, and biocompatibility, utilizing gelatin as a versatile natural scaffold material [108]. These formulation efforts were validated by a comprehensive study that investigated the preparation of topical agents derived from various Western Australian honeys (including two Manuka varieties, Jarrah, and Coastal Peppermint) compared to New Zealand Manuka [109]. The research involved transforming the honeys into standardized honey-loaded sodium alginate pre-gel solutions and subsequent wet and freeze-dried sheets to overcome dosing inconsistencies [109]. Critically, the study found that the formulation process, including the use of sodium alginate and calcium chloride, did not compromise the antioxidant activity of the honeys, which was consistently measured via FRAP and HPTLC assays across pure, pre-gel, wet, and dry sheet forms [109]. Furthermore, Australian and New Zealand Manuka, along with Jarrah honey, demonstrated consistent growth inhibition zones against all tested Gram-positive and Gram-negative bacteria. Although pure honeys generally exhibited the highest activity, the dry sheets followed closely, confirming that formulations intended for in vivo application retain the crucial antioxidant and antibacterial activities, thereby supporting their suitability as standardized topical wound healing agents [109].

#### 5.1.3. Quality Parameters of Manuka Honey

To ensure the high standard, safety, and authenticity required for a premium medicinal product, the quality of Manuka honey is governed by stringent international and national regulations. These regulations specify key physicochemical parameters, including moisture content, sugar composition, acidity, and electrical conductivity, which aim to protect consumers from deceptive practices and guarantee product integrity [97]. Two physicochemical indicators are particularly critical for quality control: diastase activity and HMF content [97]. As reported above, diastase is a key enzyme in honey, and a low level of its activity is considered an indicator of either excessive heat treatment or industrial processing, compromising the honey’s freshness [97]. Conversely, HMF, a furan compound formed from sugar degradation under acidic conditions or high temperatures, is a critical marker of quality degradation and is also used to assess potential adulteration with processed sugar additives [97]. Beyond these general quality markers, the New Zealand Ministry of Primary Industries has established a specific five-characteristic profile necessary for authenticating Manuka honey and distinguishing monofloral from multifloral varieties [97]. Four of these characteristics are specific chemical compounds, i.e., 2′-methoxyacetophenone, 2-methoxybenzoic acid, 3-phenyllactic acid, and 4-hydroxyphenyllactic acid, while the fifth involves the use of DNA markers found in Manuka pollen, providing an authoritative, multi-modal standard for authenticity [97].

#### 5.1.4. Methylglyoxal: Dual Role and Safety Considerations

The definitive component responsible for the potent, non-peroxide antibacterial activity of Manuka honey is MGO. However, MGO also presents a critical paradox, as it is a highly reactive alpha-oxoaldehyde and a potent glycotoxin widely implicated in the pathogenesis of chronic human diseases, including diabetes mellitus, neurodegenerative disorders (e.g., Alzheimer’s and Parkinson’s), and cardiovascular conditions (e.g., atherosclerosis and hypertension) [89,98]. MGO acts as a key precursor in the non-enzymatic reaction known as glycation, leading to the formation of Advanced Glycation End-products (AGEs) [89,98]. Glycation involves the reaction between the carbonyl groups of reducing sugars or dicarbonyl compounds (like MGO) and the amino groups of proteins or lipids [110]. This reaction, which proceeds through pathways such as the Maillard reaction (including the Hodge and Namiki pathways) [111,112], as well as the polyol and lipid peroxidation pathways, is a normal metabolic byproduct [111]. However, under conditions of metabolic stress or aging, the accumulation of high levels of AGEs in tissues and bloodstream induces oxidative stress and the generation of ROS [111]. Physiologically, cells possess sophisticated defense mechanisms, notably the glyoxalase system (comprising glyoxalase 1 and glyoxalase 2), which detoxifies MGO [113]. Nevertheless, when MGO synthesis exceeds this detoxification capacity, AGE accumulation occurs [113]. The subsequent binding of AGEs to their transmembrane receptors, known as Receptors for AGEs (RAGEs), initiates a cascade of intracellular events that activate inflammatory signaling pathways (e.g., NF-kB), leading to increased ROS production, inflammation, and cellular apoptosis [111].

Crucially, the potential toxicological effects of high MGO intake from Manuka honey must be balanced against the protective compounds inherently present within the honey matrix. The naturally abundant polyphenols and other antioxidants (e.g., ascorbic acid, glutathione) in honey are known to mitigate AGE damage [114]. These phenolic antioxidants exert protection through multiple mechanisms: (1) ROS scavenging: they are transformed into stable phenoxy radicals, directly attenuating oxidative stress induced by AGEs [114]; (2) chelation: their structure allows them to chelate metal ions (such as copper and iron), thereby reducing the formation of ROS [114]; (3) metabolic modulation: they can promote glucose metabolism by modulating key enzymes, such as AMP-activated protein kinase (AMPK), and activating insulin receptors [114].

This intrinsic chemical buffering capacity suggests that the consumption of Manuka honey, unlike exposure to isolated MGO, provides simultaneous administration of the glycotoxin and a powerful complex of protective and detoxifying agents, underscoring the importance of consuming the entire honey matrix rather than isolated components.

Manuka honey has attracted considerable academic and commercial interest, while research data on other honeys is much sparser. Below, the chemical composition and the bioactive properties of Jarrah and Agastache honeys, two other species that have been investigated in recent years, are discussed.

### 5.2. Jarrah Honey

Jarrah honey is a monofloral honey derived exclusively from the nectar of the *Eucalyptus marginata* tree, a towering eucalypt species endemic to the geographically isolated south-west region of Western Australia [115]. The premium status of Jarrah honey is inherently linked to the specific biology of its source, as *E. marginata* exhibits an unusually long and sporadic flowering cycle. This tree produces white or cream-colored flowers only every three to four years, a frequency that is dependent on climatic conditions [115]. This infrequent and geographically specific spring flowering, typically occurring from September to January, causes the scarcity of the resulting honey, establishing it as a highly valued medicinal commodity.

From a physicochemical perspective, Jarrah honey is characterized by a low tendency to crystallize, a property primarily attributed to its high fructose-to-glucose ratio and generally low pollen content. Reduced pollen content limits the availability of nucleation sites that normally initiate crystallization, while the elevated fructose fraction (approximately 42.5%) further stabilizes the liquid state [110]. This compositional feature is also associated with a comparatively lower glycemic index (GI) relative to many other honey varieties, which has potential nutritional and therapeutic implications [11].

#### 5.2.1. Phytochemical Profile and Chemical Markers

In recent years, analytical techniques have been crucial for defining the unique chemical signature of Jarrah honey. A 2023 study by Lawag and colleagues used high-performance thin-layer chromatography (HPTLC) to examine the phytochemical composition and antioxidant activity of Jarrah honey [116]. Compounds identified and regarded as chemical markers using this technique included hesperitin, *o*-anisic acid, taxifolin, kojic acid, *m*-coumaric acid, lumichrome, epigallocatechin gallate, and 2,3,4-trihydroxybenzoic acid. The authors sought to determine which honey constituents contribute to antioxidant activity using a novel analytical method based on hue changes upon contact with the reagent 2,2-diphenyl-1-picrylhydrazyl (DPPH) [116]. The reaction of compounds with DPPH is primarily governed by the steric accessibility of the reagent, meaning that smaller molecules have greater access to the radical site than larger molecules [116]. This explains why larger molecules like flavonoids tend to react more slowly than smaller molecules, such as simple phenolic acids [116]. The reactivity of flavonoids with DPPH is further dictated by the so-called Bors criteria, which outline specific structural requirements for enhanced antioxidant power [116]. While the HPTLC-DPPH assay is a powerful tool for identifying antioxidant constituents in vitro [116], its biological relevance is limited, as the DPPH radical does not exist naturally in biological or food systems. Therefore, the authors strongly suggest utilizing more biochemically relevant antioxidant models in future studies [116].

**Figure 2:**
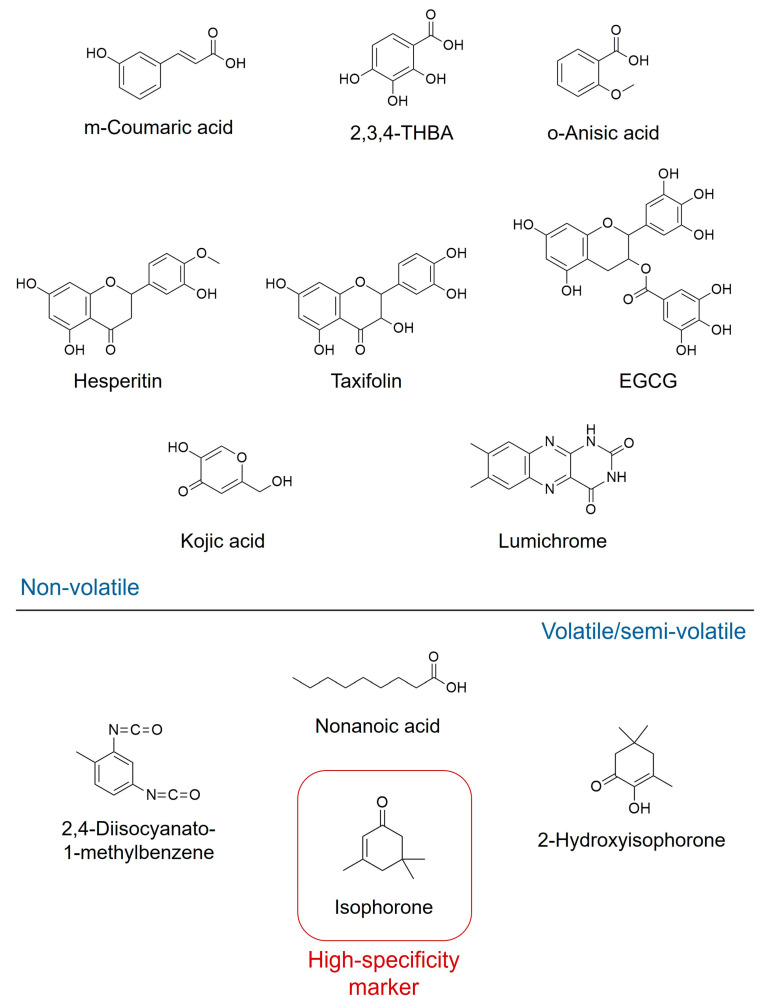
Chemical markers of Jarrah honey. 2,3,4,-THBA: 2,3,4-trihydroxybenzoic acid; EGCG: epigallocatechin gallate.

A separate study by Anand and colleagues focused on identifying the major volatile and semi-volatile bioactive compounds [117]. Their analysis showed that the dominant bioactive compounds in Jarrah honey are isophorone (40.06%), nonanoic acid (5.62%), 2-hydroxy-3,5,5-trimethyl-2-cyclohexenone (2-hydroxyisophorone; 5.12%), and 2,4-diisocyanato-1-methylbenzene (3.75%) (Figure 2) [117]. Isophorone was specifically highlighted as a unique chemical marker identified only in Jarrah honey, establishing it as a key signature compound derived from the *E. marginata* source. Nonanoic acid was also identified as a major compound, though it was additionally found in Jelly bush and Tea tree honeys, suggesting its utility as a general marker of certain floral types rather than a specific Jarrah unique marker [117].

#### 5.2.2. Antimicrobial Properties and Mechanism

On the basis of this distinctive chemical composition, Jarrah honey exhibits significant antimicrobial and antifungal activities. Its antibacterial efficacy, summarized in Table 3, is primarily associated with hydrogen peroxide (H_2_O_2_) production via the enzymatic activity of glucose oxidase, in combination with its rich phenolic profile [115]. Unlike Manuka honey, whose antimicrobial activity is largely driven by high concentrations of MGO, Jarrah honey represents a peroxide-dominant system, highlighting an important mechanistic distinction between these two medicinal honeys.

As with its antibacterial activity, while the production of H_2_O_2_ is essential, the level present in the honey appears too low to solely justify its potent antifungal activity [121]. Reported concentrations of H_2_O_2_ range from 0.04 to 4 mM, with an average value of approximately 2.5 mM. While enzymatic oxidation of glucose is the principal pathway for peroxide generation, evidence indicates that non-enzymatic mechanisms also contribute. Specifically, the autoxidation of polyphenols has been identified as a secondary source of H_2_O_2_, suggesting that total peroxide activity depends not only on enzymatic capacity but also on the concentration and qualitative composition of phenolic constituents, which act as synergizing agents [122]. This dual mechanism may partially explain the broad-spectrum antimicrobial efficacy observed for Jarrah honey.

In addition to antibacterial effects, Jarrah honey demonstrates potent antifungal activity, including activity against dermatophytes [115]. As with antibacterial action, H_2_O_2_ alone appears insufficient to fully account for this antifungal potency, indicating synergistic interactions between peroxide generation and polyphenolic compounds [121]. These findings highlight the importance of considering the integrated chemical matrix of honey, rather than attributing bioactivity to a single compound or mechanism.

#### 5.2.3. Impact on Intestinal Microbiota and Function

The intestinal microbiota has received increasing interest due to its crucial role in health. Dysbiosis, characterized by the proliferation of potentially pathogenic bacteria at the expense of probiotic species, can negatively affect intestinal immune function and barrier function, consequently leading to bowel changes. A 2020 study by Yuyuan Li and colleagues investigated the effects of Jarrah honey in a mouse model of constipation [123]. The mice were administered Jarrah honey (7.5 g/kg body weight) via gastric tube once daily for 5 days [123]. Researchers assessed fecal water content and intestinal transit rate, along with colonic concentrations of substance P (SP), vasoactive intestinal peptide (VIP), and serotonin (5-hydroxytryptamine; 5-HT) [123]. The study demonstrated that honey alleviates constipation by modulating the composition of the microbiota and promoting its eubiosis [123].

### 5.3. Agastache Honey

Agastache honey is a monofloral honey derived from *Agastache rugosa*, a perennial herbaceous plant belonging to the Lamiaceae family. Also known as Korean mint or Korean hyssop, this species is native to Australia and various parts of Asia [124].

#### 5.3.1. Chemical Signature and Authentication Markers

The distinctive chemical profile of Agastache honey is crucial for its authentication and therapeutic properties. The most abundant compounds identified include the phenylpropanoid derivative phenyllactic acid (148.3 mg/kg) and methyl syringate (46.1 mg/kg) [125]. Other phenolic compounds present in high concentrations are 4-hydroxybenzoic acid (11.8 mg/kg), *p*-coumaric acid, and gallic acid. The predominance and unique ratios of these compounds suggest their potential use as reliable chemical markers for authentication [125]. To validate the floral source, the honey’s phenolic profile was compared directly with that of *A. rugosa* flower extracts. Compounds common to both the honey and the flowers included protocatechuic acid, 4-hydroxybenzoic acid, 2,4-dihydroxybenzoic acid, chlorogenic acid, vanillic acid, caffeic acid, syringic acid, phenyllactic acid, *p*-coumaric acid, ferulic acid, sinapic acid, methylsyringate, cinnamic acid, hesperetin, and kaempferol [125]. However, notable differences were observed in the quantification of several compounds, suggesting chemical transformation or selective transfer during the nectar-to-honey process. For instance, phenyllactic acid and methyl syringate were present at very high concentrations in honey but only at smaller concentrations in the flowers. Conversely, caffeic acid and chlorogenic acid were abundant in the flowers but only detected at low concentrations in the honey [125].

**Figure 3:**
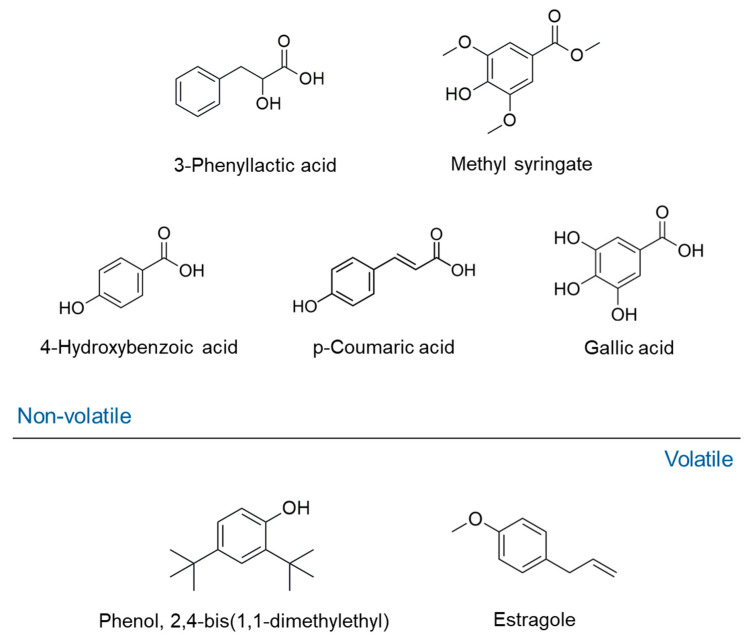
Chemical markers of Agastache honey.

In a study by Anand and collaborators (2019) [125], several volatile compounds were reported as putative markers of Agastache honey, namely phenol, 2,4-bis(1,1-dimethylethyl) (12.77% of total volatiles), estragole (12.31%), nonanoic acid, ethyl ester (7.22%), 2-propenoic acid, 3-phenyl-, ethyl ester (6.32%), hexadecanoic acid, ethyl ester (5.68%), benzaldehyde, 4 methoxy (5.17%), β-caryophyllene (4.67%), nonanal (3.19%), and 2H-benzimidazol-2-one, 1,3-dihydro-5-methyl- (2.34%). In particular, estragole and phenol, 2,4-bis(1,1-dimethylethyl) were also correlated on the antifungal activity of the same honey, as discussed in the next paragraph. This association was supported by previous data indicating the antifungal efficacy of the two compounds [117]. The chemical structure of volatile and non-volatile markers of Agastache honey are reported in Figure 3.

#### 5.3.2. Antimicrobial Mechanism and Comparative Potency

Agastache honey exhibits potent antimicrobial activity driven by a mechanism distinct from that of honeys like Manuka (Table 4). In a study by Anand and colleagues, the contribution of H_2_O_2_ to the overall antimicrobial effect was quantified [125]. While H_2_O_2_ was shown not to contribute to the activity of Manuka honey (which relies on MGO), it contributed significantly to other tested honeys, specifically 12% for Jarrah honey and 10% for Agastache honey [125]. Honeys derived from Leptospermum species (e.g., Manuka), as previously discussed, possess a primary mechanism based on the non-peroxide activity of MGO. The contribution of H_2_O_2_ to Agastache honey’s action categorizes it predominantly as a peroxide Activity honey, where the combined effect of H_2_O_2_ and its rich phenolic profile drives its therapeutic potential [125].

Agastache honey demonstrated particularly strong antifungal activity compared to both Manuka and Jarrah honeys when tested against the common dermatophytes *Trichophyton mentagrophytes* and *Trichophyton rubrum*, as well as the yeast *Candida albicans* [87]. Using agar diffusion and microdilution assays, Agastache honey was found to be effective at a concentration of 40% (*w*/*v*) against all tested fungal strains, yielding impressive zone diameters of 19.5–20 mm against the dermatophytes [87]. Crucially, the MIC and MFC values were identical at 40% against all tested strains [87]. This close equivalence between the MIC and MFC indicates a potent fungicidal action (i.e., the ability to kill the fungal cells, not just inhibit their growth). In contrast, Manuka honey only demonstrated fungistatic activity, requiring a higher 80% concentration against *T. mentagrophytes* and a 40% concentration against *T. rubrum* and *C. albicans* [87]. Jarrah honey, while showing some activity against *C. albicans*, exhibited no activity against the dermatophytes under the same assay conditions [87]. The superior antifungal efficacy of Agastache honey underscores its potential clinical utility for treating topical fungal infections [87].

### 5.4. Comparative Overview of Manuka, Jarrah, and Agastache Honeys

Manuka, Jarrah, and Agastache honeys exhibit overlapping yet distinct mechanistic profiles, bioactive compositions, and therapeutic potentials, which reflect their unique botanical and geographical origins. Manuka honey is characterized by a dominant non-peroxide mechanism, driven primarily by MGO, which confers robust antibacterial efficacy against Gram-positive and Gram-negative pathogens and underlines its clinical use in wound management. Conversely, Jarrah and Agastache honeys rely mainly on H_2_O_2_-mediated activity, with phenolic compounds acting synergistically to enhance antimicrobial and antifungal effects. While Jarrah shows broad-spectrum antibacterial activity and moderate antifungal potential, Agastache demonstrates a potent fungicidal activity, notably against dermatophytes and *C. albicans*, highlighting its potential for topical antifungal applications.

Despite these differences, all three honeys share bioactive convergence, including phenolic and flavonoid constituents that contribute to antioxidant, anti-inflammatory, and immunomodulatory effects. However, the relative contribution of individual components differs: MGO dominates in Manuka, specific polyphenols and unique volatiles in Jarrah, and phenyllactic acid, methyl syringate, and estragole in Agastache. These differences result in distinct chemical fingerprints that can serve both for authentication and mechanistic inference.

From a translational perspective, these mechanistic distinctions carry important implications for therapeutic applications. The MGO-driven activity of Manuka honey allows for consistent antibacterial performance across diverse wound microbiomes, while Jarrah and Agastache honeys may exhibit variable activity depending on H_2_O_2_ production and phenolic composition, which are influenced by floral source, harvest season, and storage. The pronounced antifungal potency of Agastache honey, despite modest peroxide contribution, underscores the role of specific phenolic and volatile constituents in augmenting efficacy, suggesting potential for development as a targeted antifungal agent.

## 6. Security-Related Issues

Globally, contamination by trace elements has declined due to more effective control of pollutant emissions and the implementation of stricter environmental regulations. Nevertheless, environmental pollution remains a persistent and significant public health concern. Urban and natural ecosystems continue to be threatened by industrial activities that are economically indispensable yet inherently generate substantial environmental contamination [126]. Laboratory studies have highlighted the adverse effects of metal and metalloid exposure on Western honeybees. At a historic mining site, bees were exposed to pollutants through dust, food, and water. More than 1000 bees collected from five apiaries located along an 11 km contamination gradient near a former gold mine in southern France were analyzed. The bees closest to the mine exhibited a 36% reduction in olfactory learning performance, and three-dimensional brain imaging revealed a 4% reduction in the volume of their olfactory centers, suggesting neurodevelopmental impairments linked to contaminant exposure [127,128].

During foraging, bees can inadvertently gather environmental contaminants such as pesticides (herbicides, fungicides, and insecticides) along with nectar. Since honey reflects the chemical characteristics of the surrounding environment, it serves as a valuable indicator of overall ecosystem health. Pesticide residues detected in honey can be evaluated in relation to their potential impact on human health using maximum residue limits (MRLs). An MRL, expressed in milligrams of residue per kilogram of food, represents the highest permissible concentration of a pesticide considered legally acceptable by the Codex Alimentarius Commission for foods and feeds [129]. Human exposure to the herbicide glyphosate and its metabolite aminomethylphosphonic acid (AMPA) has been associated with metabolic disturbances and oxidative stress [130,131]. Epidemiological research has linked glyphosate exposure to several health disorders, including cancer [132], respiratory diseases [133], chronic kidney disease [134], neurological disorders, as well as metabolic dysfunction and oxidative stress. In the European Union, the MRL for glyphosate in honey is set at 0.05 ppm [135], whereas the New Zealand Ministry of Primary Industries allows a higher maximum value of 0.1 ppm, a discrepancy that has generated significant controversy. Recent reports highlight the detection of glyphosate above the legal limits in several honey samples from EU [136]; regarding Manuka honey from New Zealand, a recent independent has detected glyphosate residues above the National limits in some retail samples (https://nomoreglyphosate.nz/glyphosate-in-nz-honey-first-test-results/, accessed on 27 November 2025), although this has not been yet documented in the scientific literature. Nevertheless, these data indicate that the development of more rigorous and harmonized glyphosate regulations remains essential.

Heavy metal contamination in honey may originate from environmental pollution, certain beekeeping and agricultural practices, metal containers used during processing, and physicochemical characteristics of the soil and water at the honey’s geographical origin. The most frequently detected heavy metals in honey include cadmium, lead, and arsenic [89]. Regulatory limits set maximum concentrations of 0.10 mg/kg for lead and 0.05 mg/kg for cadmium in honey [135], while the tolerable daily intake of arsenic is 3.0 μg/kg of body weight [137].

Hydroxymethylfurfural (HMF) is a cyclic aldehyde formed from the degradation of hexoses during prolonged storage, a process accelerated under acidic conditions, elevated temperatures, or via the Maillard reaction. Although not unique to honey, HMF is widespread in heat-processed foods containing sugars. The physicochemical properties of honey, including pH, acidity, fructose-glucose ratio, mineral content, and water activity, favor its formation. Since fresh honey contains little to no HMF, its concentration is widely used as an indicator of freshness and processing intensity. Storage conditions and the use of metal containers also influence HMF formation; therefore, minimizing heat exposure and storage duration is recommended [59]. To ensure product safety and quality, the Codex Alimentarius Commission has established a maximum HMF limit of 40 mg/kg for honey, with an increased limit of 80 mg/kg for honeys produced in tropical regions. Excessive intake of HMF has been linked to mutagenic, genotoxic, and organotoxic effects, with the compound demonstrating enzyme-inhibitory and DNA-damaging potential in multiple in vitro, in vivo, and preclinical studies [59]. After ingestion, HMF is readily absorbed from the gastrointestinal tract and subsequently metabolized into several derivatives excreted in urine. Interestingly, while HMF is associated with harmful effects, its metabolite 5-sulfoxymethylfurfural also exhibits beneficial biological activities, including antioxidant, antiallergic, anti-inflammatory, antihypoxic, anti-sickling, and antihyperuricemic properties. Following oral or intravenous administration, HMF is primarily converted into three metabolites [5-hydroxymethylfuroic acid (HMFA), 2,5-furandicarboxylic acid (FDCA), and 5-(hydroxymethyl)-2-furoyl glycine (HMFG)] and may also form a fourth product, 5-sulfoxymethylfurfural (SMF) [138]. In bees, HMF is suspected to cause dysentery-like symptoms and gastrointestinal ulceration, ultimately contributing to mortality [139]. HMF content in commercial manuka and jarrah honeys from Oceania shows considerable variability, reflecting differences in storage and processing practices [110].

Pyrrolizidine alkaloids (PAs) and their N-oxides (PANOs) are secondary metabolic compounds produced by plants as chemical defenses against herbivores. They are classified into four groups based on the structure of their necine base, i.e., platynecine, retronecine, heliotridine, and otonecine. PAs are typically not found in free base form but occur as mono-, di-, or macrocyclic diesters formed by the esterification of a necine base with one or more aliphatic mono- or dicarboxylic acids, contributing to their structural diversity. The core pyrrolizidine skeleton consists of two fused saturated five-membered rings bridged by a nitrogen atom, and the presence of a 1,2-double bond often enhances toxicity. In their native form, PAs are protoxins and biologically inactive. To exert toxicity, they require metabolic activation, primarily in the liver, where CYP450 monooxygenases convert them into reactive electrophilic metabolites such as 6,7-dihydro-7-hydroxy-1-(hydroxymethyl)-5H-pyrrolizine. These metabolites readily bind to nucleophilic sites in DNA, proteins, and amino acids, forming pyrrole adducts that may persist in tissues and induce toxicity, particularly in the liver. PAs have been shown to possess hepatotoxic, pneumotoxic, genotoxic, carcinogenic, and developmental toxic effects. Detoxification can occur through conjugation with glutathione, producing more soluble and less toxic metabolites that are more easily excreted [135]. Recent studies in Australia have documented the presence of PAs in monofloral and multifloral honeys, including those produced by stingless bees. UHPLC-MS/MS analyses detected PAs such as lycopsamine, indicine, and intermedine, with plant origins traced to Parsonsia spp. and Ageratum conyzoides near apiaries [140]. These findings highlight that PA contamination remains a real safety concern even for premium medicinal honeys in Oceania. Exposure to PAs is considered one of the primary causes of hepatic sinusoidal obstruction syndrome (HSOS), formerly known as hepatic veno-occlusive disease, a rare but severe hepatic vascular disorder associated with high mortality [141,142].

## 7. Issues Related to Adulteration and Counterfeiting

Food fraud is a major concern for high-value medicinal honeys from Oceania, particularly Manuka and Jarrah. Because these honeys command premium prices and are prized for their therapeutic properties, they are especially attractive targets for adulteration and counterfeiting. In this context, fraud does not simply erode economic value, since it can undermine the bioactive properties that makes these honeys “medicinal.” The most common form of adulteration involves the addition of inexpensive sugar syrups such as glucose, rice, corn, or maple syrup, to bulk up the product. Scientific studies have directly demonstrated this in Australian monofloral honeys. For example, using HPTLC, researchers successfully detected the addition of several syrup types (rice, corn, golden, maple, treacle) even at relatively low adulteration levels in both Manuka and Jarrah honeys [143]. Their chemometric analysis, combining sugar-profile and organic-extract data, allowed robust discrimination between authentic and adulterated samples [138].

Counterfeiting, in contrast, often involves misrepresentation of the botanical or geographic origin of honey, or the creation of synthetic honey analogues. Authenticating floral origin is critical for medicinal honeys because their value hinges on specific chemical markers. For instance, the recent article by Lawag and collaborators reporting the “nectar signature” for Jarrah honey that persist in the finished product provide a valuable tool to verify true Jarrah origin [116]. Despite these advances, longstanding analytical challenges remain, especially for manuka honey. The widely used AOAC 998.12 method (a stable carbon isotope test) can produce false positives in genuine Manuka. This is due to the conversion of DHA, naturally present in manuka nectar, to MGO over time or during storage, a reaction that shifts the δ^13^C of the protein fraction and mimics the isotopic signature of C_4_-sugar adulteration. Classic work has shown that insoluble components like pollen co-precipitate during protein isolation in this test, which further skews the δ^13^C and may lead to misclassification. Adding to the challenge, pollen-based authentication (melissopalynology) can also be problematic. In Jarrah honey, for example, there is evidence that pollen analysis may misidentify closely related *Eucalyptus* species, making it unreliable as a sole indicator of floral origin. This underscores the need for combined chemical and botanical approaches.

More recently, researchers have moved toward multi-marker and chemometric techniques. The HPTLC-chemometric approach described earlier allowed discrimination of adulterated manuka and jarrah honeys even at low syrup addition percentages by using principal component analysis (PCA), cluster analysis, and artificial neural networks (ANN) [139,144,145]. These methods are particularly promising for defending medicinal honeys from fraud because they do not rely on just sugar composition but also track the organic extract fingerprint. Furthermore, elemental profiling has emerged as another powerful strategy. By analyzing the mineral content of honey and comparing it with known syrups, sPLS-DA (sparse Partial Least Squares Discriminant Analysis) models have achieved high accuracy in distinguishing pure honeys from adulterated ones [146,147]. This mineral-chemometric method could provide a stable and robust approach to detect syrup adulteration in a way that is less vulnerable to manipulation by counterfeiters.

The implications of adulteration and counterfeiting for medicinal honey integrity are profound. If a Manuka or Jarrah honey is diluted or mislabeled, its characteristic bioactive constituents may be reduced or masked, potentially diminishing therapeutic efficacy. To preserve the integrity of these medicinal honeys, research and industry must continue to develop and implement authentication frameworks. These should integrate isotopic methods, chemometric fingerprinting, nectar-marker profiling, and elemental analysis. Equally important is establishing regional reference libraries of authenticated honeys (with known botanical and geographic origin) to serve as benchmarks.

## 8. Conclusions

In recent years, honey has attracted considerable scientific interest for its potential beneficial properties, particularly anti-inflammatory, antimicrobial, antioxidant, and wound-healing activities, building on its traditional nutritional and medicinal use. Among honeys from Oceania, Manuka is by far the most extensively studied, with robust in vitro, preclinical, and some clinical evidence supporting its bioactivity and therapeutic potential. However, this preponderance of research has created a literature bias, leaving other medicinal honeys from the region, such as Jarrah and Agastache, comparatively under-characterized. Many endemic honeys remain unexplored, and their chemical composition, bioactive constituents, and potential clinical applications are largely unknown.

A critical limitation in the current literature is the high variability in reported bioactivities, reflecting differences in floral origin, geographical location, harvesting, and processing conditions. Standardization in honey characterization is frequently lacking, making comparisons between studies difficult and complicating efforts to translate in vitro findings into clinical practice. Moreover, the majority of antimicrobial and antifungal studies rely on planktonic bacterial models and high honey concentrations in vitro, which do not fully capture the complexity of human infections, particularly biofilm-associated or chronic wounds. The bioavailability of bioactive compounds at the tissue interface, as well as the impact of formulation (e.g., medical-grade honey, hydrogels, or dressings), are often overlooked, limiting our understanding of how laboratory results can be applied clinically.

Clinical evidence remains sparse, with most studies conducted in vitro or in preclinical models. This gap emphasizes the need for well-designed human trials to validate efficacy and safety, particularly for honeys beyond Manuka. Additionally, synergistic effects with conventional treatments, dosage optimization, and long-term safety profiles have not been systematically evaluated, further limiting clinical translation.

Future research should prioritize under-represented honeys, with comprehensive chemical and bioactivity profiling using standardized methods. Investigations should aim to uncover the molecular mechanisms responsible for observed bioactivities and assess their therapeutic potential in clinical settings. By addressing these gaps, researchers can expand the medicinal relevance of Oceania honeys, moving beyond the well-studied Manuka to fully exploit the region’s rich apicultural biodiversity. Ultimately, such efforts could pave the way for novel natural therapeutics with well-defined efficacy, safety, and health-promoting properties.

Finally, as market demand increases, a greater tendency toward fraud has become prevalent, leading to the use of analytical tools for quality assessment. It is therefore essential to pay attention to honey’s authenticity, seeking to develop techniques that can reliably determine its botanical origin to identify cases of adulteration and counterfeiting.

## Figures and Tables

**Table 1:** Average chemical composition of honey per 100 g.

Proximate	g/100 g	Minerals	mg/100 g	Vitamins	mg/100 g
Fructose	38.2	Potassium	40.0–3500.0	Ascorbic acid	2.2–2.5
Glucose	31.3	Calcium	3–31	Niacin	0.1–0.2
Water	17.1	Phosphorus	2.0–15.0	Pantothenic acid	0.02–0.11
Other disaccharides	5.0	Sodium	1.6–17.0	Pyridoxine	0.01–0.32
Sucrose	0.7	Magnesium	0.7–13.0	Riboflavin	0.01–0.02
Organic acids	0.5	Zinc	0.05–2.00	Thiamin	0.0–0.01
Proteins, amino acids	0.3	Iron	0.03–4.00		
		Manganese	0.02–2.0		
		Copper	0.02–0.60		
		Selenium	0.001–0.003		

**Table 2:** Exemplificative data showing the antibacterial activity of Manuka honey.

Targeted Microbe	Efficacy	Active Constituents	Ref.
*Staphylococcus aureus*	MIC: 1.1 mM (MGO) MIC: 4.3 mM (GO)	MGO/GO	[82]
*Escherichia coli*	MIC: 1.1 mM (MGO) MIC: 6.9 mM (GO)	MGO/GO	[82]
*Pseudomonas aeruginosa*	MIC: 12% *w*/*v* MBC: 16% *w*/*v*	N/R	[83]
*Salmonella enterica*	MIC = 8.9% (*v*/*v*)	N/R	[84]
*Pseudomonas aeruginosa* (ATCC 27853)	MIC = 9.5% (*w*/*v*); MBC = 12% (*w*/*v*)	N/R	[91]
*Pseudomonas aeruginosa* (biofilm)	MIC: 5–7% *w*/*v*; MBC: 15% *w*/*v*	N/R	[92,93]
*E. coli*, *Salmonella typhimurium*, *Salmonella enteritidis*, *Salmonella mississippi*, *Yersinia enterocolitica*, *Enterobacter aerogenes*, *Enterobacter cloacae*, *Shigella flexneri*, *Shigella sonnei*	MIC: 5–10% (*v*/*v*); MBC: 5–10% (*v*/*v*); *Enterobacter* spp. MIC/MBC = 10–17%	H_2_O_2_, MGO	[93]
*Staphylococcus aureus*	MIC_50_: UMF 5+ = 6%, UMF 10+ = 7%, UMF 15+ = 15%; for Enterobacteriaceae, MIC_50_ ~21–27%	MGO	[94]
*Bacillus subtilis*	MIC = 0.8 mM	MGO	[95]
*Escherichia coli*	MIC = 1.2 mM	MGO	[95]

UMF: Unique Manuka Factor: based on the measured level of MGO. UMF 5+: ≥83 mg/kg, UMF 10+: ≥263 mg/kg, UMF 15+: ≥514 mg/kg. MGO: methylglyoxal.

**Table 3:** Exemplificative data showing the antibacterial activity of Jarrah honey.

Targeted Microbe	Efficacy	Active Constituents	Ref.
*Staphylococcus aureus*	MIC: 4–12% *w*/*v*; MIC_50_: 4–8% *w*/*v*	H_2_O_2_	[118]
Methicillin-resistant *Staphylococcus aureus* (MRSA)	MIC: 6–12% *w*/*v*	H_2_O_2_	[118]
Coagulase-negative staphylococci	MIC_50_: 4–8% *w*/*v*	H_2_O_2_	[118]
*Streptococcus pyogenes*	MIC: 8–29% *w*/*v*	N/R	[119]
*Enterococcus faecalis*	MIC: 10–25% *w*/*v*	N/R	[119]
*Escherichia coli*	MIC: 5% *w*/*v*	N/R	[119]
*Pseudomonas aeruginosa*	MIC: 5–16% *w*/*v*	N/R	[82]
*Staphylococcus epidermidis*	MIC: 4–12% *w*/*v*	N/R	[120]
*Candida albicans* (ATCC 10231)	MIC: 20% *w*/*v*	N/R	[120]
Dermatophyte fungi (*Trichophyton rubrum*, *T. mentagrophytes*)	MIC: 1.5–3.5% *w*/*v*	H_2_O_2_	[121]
*Microsporum canis*	MIC: 1.5–3.5% *w*/*v*	H_2_O_2_	[121]
Other Gram-negative clinical isolates (general panel)	MIC: 6.7–28.0%	H_2_O_2_	[121]

The ranges of MIC are related to different Jarrah samples tested within the same study. N/R: not reported.

**Table 4:** Exemplificative data showing the antibacterial activity of Agastache honey.

Targeted Microbe	Efficacy	Active Constituents	Ref.
*Staphylococcus aureus* (MSSA ATCC-25923)	MBC ≈ MIC: 6–25% *w*/*v*	Phenyllactic acid, methyl syringate; total phenolics	[125]
*Staphylococcus aureus* (MRSA ATCC BAA-1698)	MBC ≈ MIC: 6–25% *w*/*v*	Phenyllactic acid, methyl syringate; total phenolics	[125]
*Escherichia coli* (ATCC-11560 or clinical isolate)	MBC ≈ MIC: 6–25% *w*/*v*	H_2_O_2_; total phenolics	[125]
*Pseudomonas aeruginosa* (ATCC 21853 or clinical isolate)	MBC ≈ MIC: 6–25% *w*/*v*	H_2_O_2_; total phenolics	[125]
*Trichophyton mentagrophytes*	MFC: 40% *w*/*v*; zone diameter ~20 mm	H_2_O_2_; volatile terpenes, mainly estragole and phenol-2,4-bis (1,1-dimethylethyl)	[117]
*Trichophyton rubrum*	MFC: 40% *w*/*v*; zone diameter: 19.5 mm	H_2_O_2_; volatile terpenes, mainly estragole and phenol-2,4-bis (1,1-dimethylethyl)	[117]
*Candida albicans* (ATCC 10231 + clinical isolate)	MFC: 40% *w*/*v*	H_2_O_2_; volatile terpenes, mainly estragole and phenol-2,4-bis (1,1-dimethylethyl)	[117]

The ranges of MIC are related to different Agastache samples tested within the same study.

## Data Availability

No new data were created or analyzed in this study.
